# Remarkable alterations of Nav1.6 in reactive astrogliosis during epileptogenesis

**DOI:** 10.1038/srep38108

**Published:** 2016-12-01

**Authors:** Hongyan Zhu, Yuxiao Zhao, Hao Wu, Nan Jiang, Ziyi Wang, Weide Lin, Jiahui Jin, Yonghua Ji

**Affiliations:** 1 Laboratory of Neuropharmacology and Neurotoxicology, School of Life Science, Shanghai University, Nanchen Road 333, Shanghai, 200444, China

## Abstract

Voltage-gated sodium channels (VGSCs) play a vital role in controlling neuronal excitability. Nav1.6 is the most abundantly expressed VGSCs subtype in the adult central nervous system and has been found to contribute to facilitate the hyperexcitability of neurons after electrical induction of status epilepticus (SE). To clarify the exact expression patterns of Nav1.6 during epileptogenesis, we examined the expression of Nav1.6 at protein and mRNA levels in two distinct animal models of temporal lobe epilepsy (TLE) including a post-SE model induced by kainic acid (KA) intrahippocampal injection and a kindling model evoked by pentylenetetrazole (PTZ). A prominent, seizure intensity-dependent increase of Nav1.6 expression in reactive astrocytes was observed in ipsilateral hippocampus of post-SE rats, reaching the peak at 21 days after SE, a time point during the latent stage of epileptogenesis. However, Nav1.6 with low expression level was selectively expressed in the hippocampal neurons rather than astrocytes in PTZ-kindled animals. This seizure-related increase of a VGSCs subtype in reactive astrocytes after SE may represent a new mechanism for signal communication between neuron and glia in the course of epileptogenesis, facilitating the neuronal hyperexcitability.

Epilepsy is one of the most common neurological diseases with a prevalence of 0.5–2% of the population worldwide, which is characterized by the periodic and unprovoked occurrence of seizures that manifest neuronal hypersynchrony and hyperexcitability^1^. Although seizures in many patients with epilepsy can be well-controlled with currently available antiepileptic drugs (AEDs), a substantial proportion (~30%) of patients who do not respond to any AEDs will ultimately develop intractable epilepsy^2^.

Voltage-gated sodium channels (VGSCs) play a vital role in controlling neuronal excitability as they are essential for the initiation and propagation of action potentials. VGSCs are protein complexes consisting of a main structural component called α-subunit forming the ion conducting pore as well as the channel gate for activation and inactivation, and four auxiliary β-subunits modulating the gating kinetics of α-subunits. In mammals, a family of 10 α-subunit genes have been cloned and encode Nav1.1–Nav1.9, Nax. Of the multiple isoforms of VGSCs, Nav1.1, Nav1.2, Nav1.3 and Nav1.6 are predominantly expressed in the brain, each of which has distinct distribution and functional characteristics^3^,^4^. Nav1.3 is primarily expressed in embryonic and neonatal brain, whereas Nav1.1, Nav1.2, and Nav1.6 are highly expressed in adult brain^4^. Increasing evidences demonstrate that the abnormal expression or function of VGSCs leading to neural network hyperexcitability could be associated with the generation of seizure activities^5^,^6^. In humans, mutations in the genes encoding these VGSC subtypes have been found in individuals with genetic epilepsy syndromes with a wide range of severity^7^. SCN1A encoding Nav1.1 has been shown to be the most frequent target of mutations and is responsible to a variety of epilepsy syndromes^8^. Recently, much attention has been paid to SCN8A encoding Nav1.6 involved in the pathogenesis of epilepsy^9^,^10^,^11^,^12^,^13^,^14^,^15^,^16^.

Nav1.6 is the most abundantly expressed sodium channel in the adult central nervous system, which is distributed at the cell body, axon initial segment (AIS) and nodes of Ranvier in both excitatory and inhibitory neurons^17^,^18^. Nav1.6 contributes to the production of tetrodotoxin-sensitive (TTX-S) transient current, persistent current, and resurgent current^19^. Several mutations of SCN8A have been recently identified and have been found to be functionally associated with epilepsy syndrome^9^,^10^,^11^,^16^,^20^. It has been found that Nav1.6 plays a role in facilitating the hyperexcitability of medial entorhinal cortex layer II neurons at 7 days after electrical induction of status epilepticus^12^. Moreover, recent studies demonstrate that Nav1.6 is a key determinant of neuronal network hyperexcitability and spontaneous epileptiform activity in animal models of Alzheimer disease (AD)^21^,^22^. The expression of Nav1.6 has been reported to be persistently reduced during epileptogenesis in post status epilepticus (SE) animals induced by pilocarpine and kainic acid^13^,^14^. However, it is also shown that Nav1.6 expression is increased both in electrical kindled-animals and post-SE animal induced by electrical stimulations^12^,^15^. Thus, the exact alterations of Nav1.6 during epileptogenesis are required to be thoroughly clarified.

Animal models of temporal lobe epilepsy (TLE) can mimic many pathological key aspects of chronic TLE in humans and are regarded as a useful tool in the study of epileptogenesis^23^. In this study, the expression patterns of Nav1.6 were determined by using two distinct animal models of TLE including post-SE model induced by kainic acid (KA) intrahippocampal injection and kindling model evoked by pentylenetetrazole (PTZ). We found that the expression of Nav1.6 in the hippocampus after SE was remarkably increased in reactive astrocytes rather than neuron or other glial cells during epileptogenesis, which is related to the severity of SE induced KA. However, the remarkable increase of Nav1.6 expression in astrocytes was not observed in the kindling animals. These findings suggest that the increased expression of Nav1.6 is an important molecular change in the progression of reactive astrogliosis during epileptogenesis.

## Results

### Seizure activities induced by KA and PTZ

Kainic acid (0.5 μg) was injected into the CA3 subarea of the right dorsal hippocampus in rats to induce status epilepticus. Behaviorally, SE was characterized by continuous limbic seizures which consisted of head bobbing, wet dog shakes and rearing that started 19.6 ± 1.35 min after intrahippocampal kainic acid injection. There were 14 rats died during or shortly after SE. Eight rats did not develop SE but only showed intermittent stage 1–3 seizure activity for about 30–60 min. Spontaneous recurrent seizures (SRS) in rats cannot be observed at 1 week after SE. Moreover, the rats that underwent SE all exhibited SRS during a period of 12–63 days after KA injection.

The seizure activities at stage 1–2 seizure in PTZ injected rats were firstly o bserved on day 6. The generalized tonic–clonic convulsions were then evoked by PTZ administration on day 15. After continuous PTZ injection for 28 days, the rats (n = 20) exhibited three subsequent stage 4 seizures after PTZ induction which is defined as successfully kindled rats. Five rats died before the termination of the experiment. The rats with PTZ administration for 1 day, 7 days and 28 days, respectively, were sacrificed for quantitative real-time PCR and immunofluorescence analysis.

### Dynamical increase of Nav1.6 in the hippocampus of KA induced post-SE models

**Figure 1:**
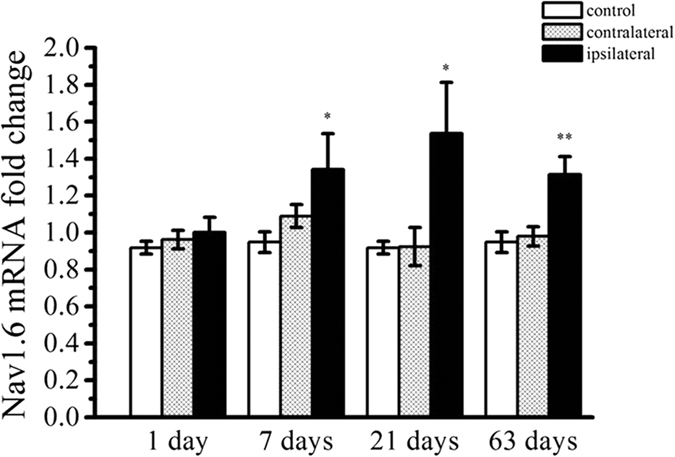
Real-time quantitative PCR analysis for Nav1.6 expression in the hippocampus in KA-induced post-SE rats and the control groups. Each column represents mean ± SEM of five animals in each group. *P < 0.05, **P < 0.01, significantly different from the control animals.

Quantitative real-time PCR was performed to examine Nav1.6 gene expression in the KA induced post-SE rats. The Nav1.6 expression at mRNA levels of the ipsilateral hippocampus in the rats experiencing SE was significantly higher than that of the contralateral and the control hippocampus at 7, 21, 63 days post-SE (*P *< 0.05, respectively) (Fig. 1).

**Figure 2:**
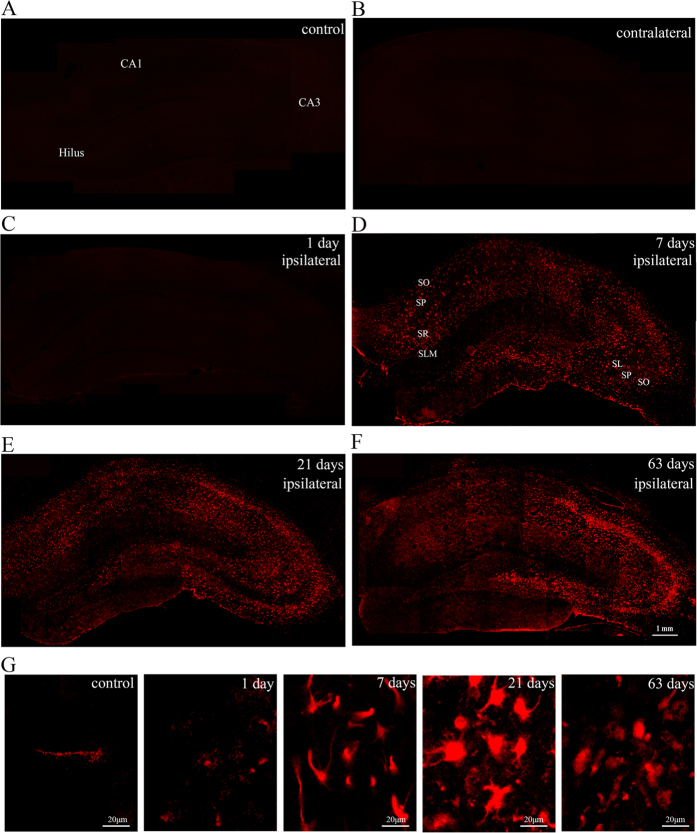
Photomicrographs of Nav1.6 immunoreactivity in the hippocampus in KA-induced TLE rats and the control groups. (**A–F**) Nav1.6-ir (red) is progressively increased in the ipsilateral hippocampus at 1 day, 7 days, 21 days, 63 days after SE, peaking at 21 days after SE compared with the contralateral side and the controls. (**G**) Nav1.6-ir (red) positive cells present morphological changes with the development of epilepsy. SO: stratum oriens; SR: stratum radiatum; SL: stratum lucidum; SP: stratum pyramidale.

**Figure 3:**
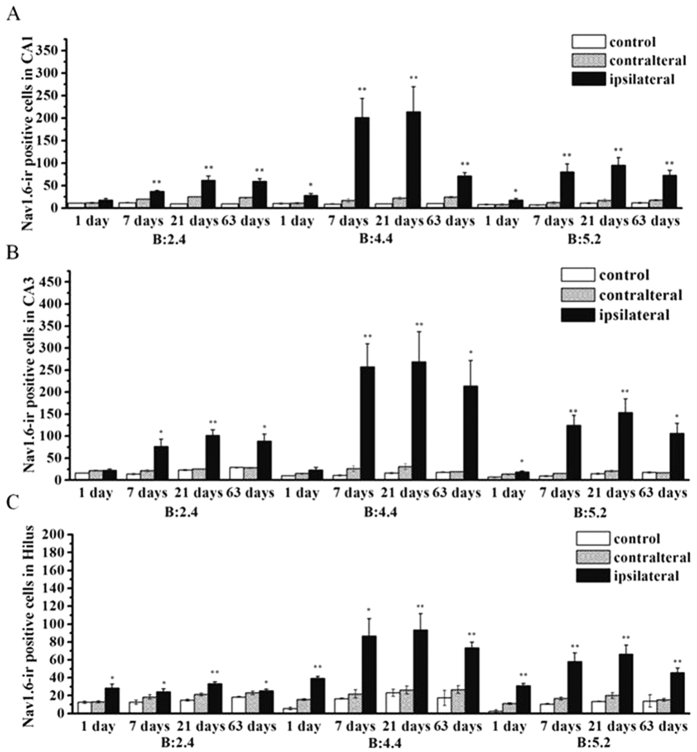
Stereological analysis of Nav1.6-ir positive cell numbers in the hippocampus in KA-induced TLE rats. There are significant increases in the number of Nav1.6-ir positive cells in the CA1 (**A**), CA3 (**B**) and hilus (**C**) subregions of the ipsilateral hippocampus at 1 day, 7 days, 21 days, and 63 days after SE in KA-induced TLE rats in comparison with that of the control group and the contralateral side. *P < 0.05, **P < 0.01, significantly different from the control animals.

**Figure 4:**
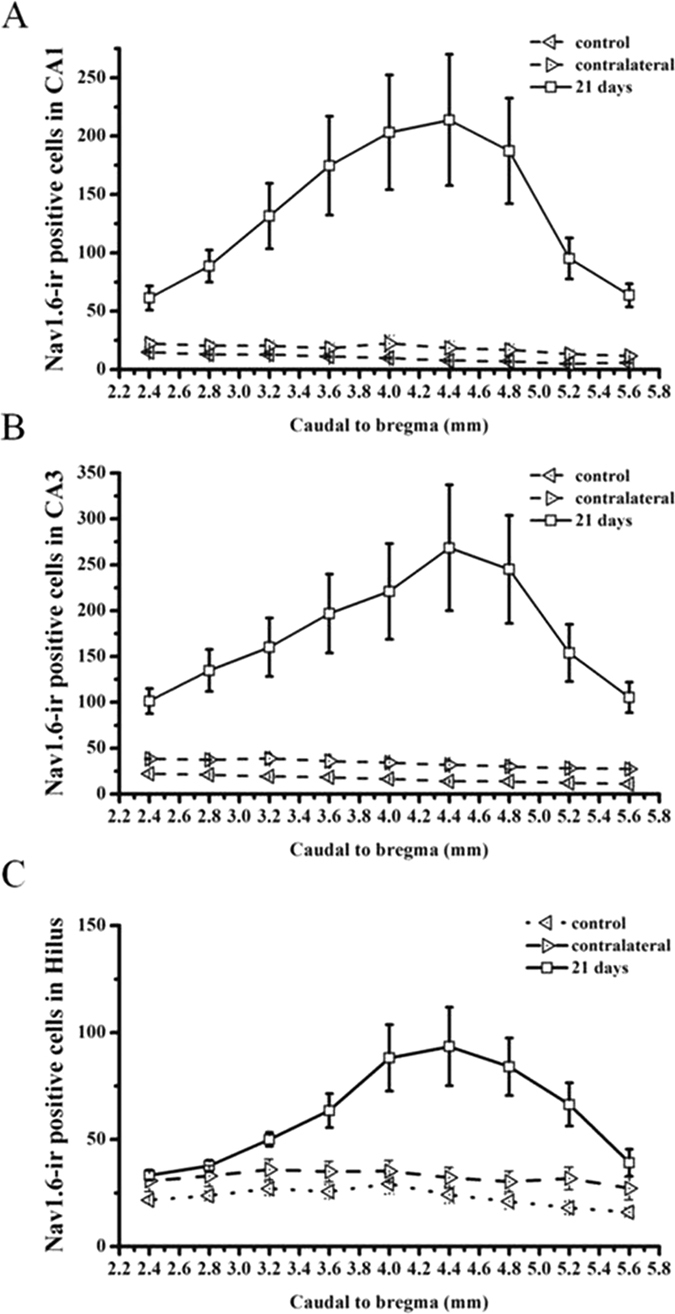
Topographical expression of Nav1.6 in the CA1 (**A**), CA3 (**B**) and hilus (**C**) subregions of the ipsilateral hippocampus (from 2.4 to 5.6 mm from bregma along the rostro-caudal axis) in KA-induced TLE rats at 21 days after SE. Note that there are more Nav1.6-ir positive cells in the sections near the KA injection site (4.0 mm caudal to bregma). Data are plotted as mean ± SEM of five animals in each group. *P < 0.05, **P < 0.01, significantly different from the control animals.

In this investigation, immunofluorescence was used to examine the expression and distribution of Nav1.6 in KA induced post-SE rats and the control groups. In the control groups, a small amount of Nav1.6 immunoreactivity (ir) was observed in the CA region and hilar region of the hippocampus (Fig. 2A). In KA injected rats without SE, there was no significant difference in the number of Nav1.6-ir positive cells in the ipsilateral hippocampus compared to the contralateral and the control hippocampus (P > 0.05), whereas the rats experiencing SE showed various degrees of Nav1.6-ir increase in the ipsilateral hippocampus compared with the contralateral hippocampus and the control hippocampus (P < 0.05)(Fig. 3A–C). In rats experiencing SE, Nav1.6-ir was persistently increased in stratum oriens (SO), stratum pyramidale (SP), stratum radiatum (SR) and stratum lucidum (SL) of the CA region, and the hilus in the ipsilateral hippocampus at 1, 7, 21, 63 days after SE, reaching the peak at 21 days after SE compared with the controls (Fig. 2B–F). Additionally, the number of Nav1.6-ir positive cells was relatively large in sections (from 3.5 mm to 4.5 mm caudal to bregma) near the KA injection site (4.0 mm caudal to bregma) of the ipsilateral hippocampus, especially in the rats experiencing more than 4 stage 5 seizures at 21 days after SE (Fig. 4A–C).

**Figure 5:**
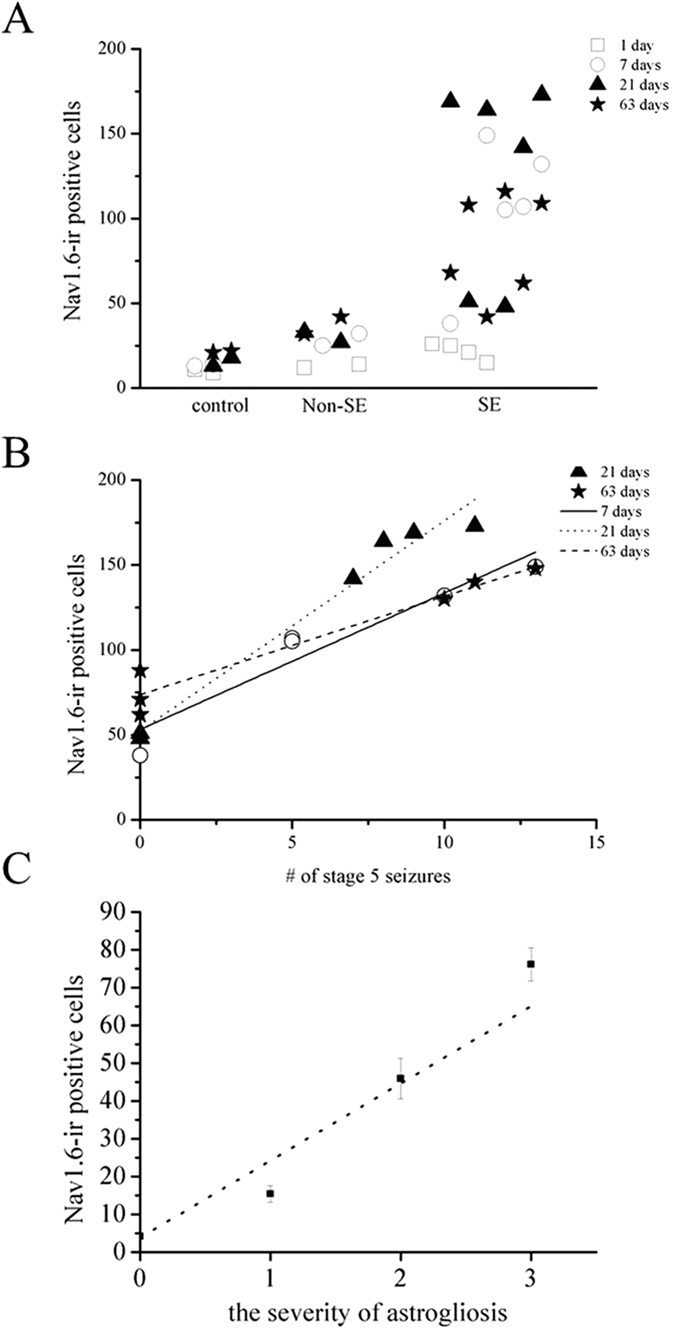
(**A**) The number of Nav1.6-ir positive cells in the ipsilateral hippocampus was closely correlated with the onset of SE in rats with KA intrahippocampal injection (R^2^ = 0.754, *P* < 0.01). (**B**) Regression analysis demonstrating the strong relationship between the number of Nav1.6-ir positive cells in the ipsilateral hippocampus and the severity of SE estimated by the number of stage 5 seizures for rats experiencing SE at 7 days, 21 days and 63 days after SE (at 7 days after SE, R^2^ = 0.975 *P* < 0.01; at 21 days after SE, R^2^ = 0.986, *p* < 0.01; at 63 days after SE, R^2^ = 0.941, *P* < 0.01). (**C**) The number of Nav1.6-ir positive cells in the ipsilateral hippocampus was profoundly correlated with the severity of astrogliosis in post-SE rats (R^2^ = 0.899).

Moreover, the increase of Nav1.6-ir was closely correlated with the onset of SE (R^2^ = 0.754, *P* < 0.01) (Fig. 5A). Furthermore, there was strong correlation between the number of Nav1.6-ir positive cells and the severity of SE assessed by the number of stage 5 seizures occurring during SE (at 7 days after SE, R^2^ = 0.975, *P* < 0.01; at 21 days after SE, R^2^ = 0.986, *P* < 0.01; at 63 days after SE, R^2^ = 0.941, *P* < 0.01) (Fig. 5B). In addition, in two rats with severe SE at 21 days after SE, we also observed the increased Nav1.6-ir in the contralateral hippocampus, but where the amount of Nav1.6-ir was littler than that of the ipsilateral hippocampus.

Besides the remarkable alteration in Nav1.6-ir positive cell numbers, Nav1.6-ir positive cells exhibited morphological changes with the course of time as the size of cell body dramatically enlarged at 21, 63 days after the induction of seizures. The majority of Nav1.6-ir positive cells were seen to exhibit fusiform and polygon-shaped morphology at 21, 63 days after SE while most of Nav1.6-ir positive cells in 24 h post-SE rats showed a typical stellate-shape (Fig. 2G).

### The localization of increased Nav1.6 in reactive astrocytes in KA induced post-SE animals

**Figure 6:**
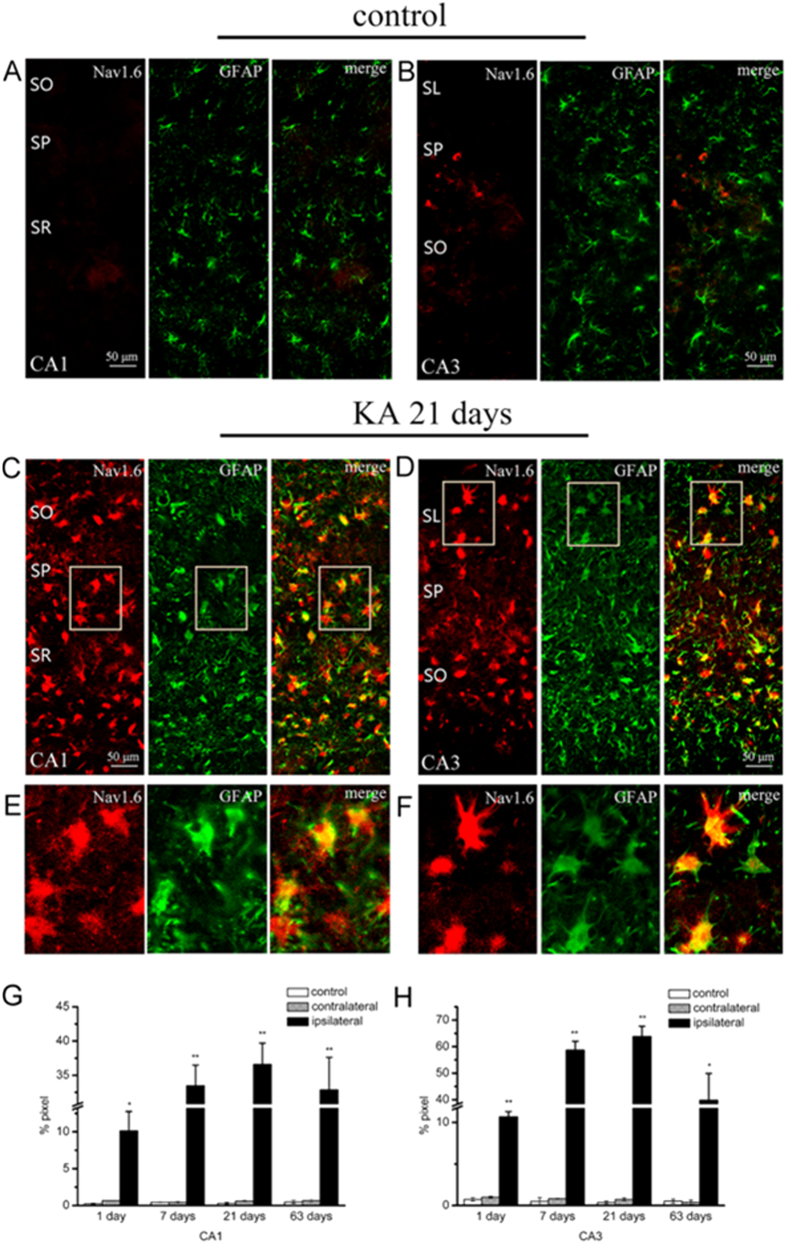
Colocalization of Nav1.6 (red) with GFAP (green) in the hippocampus in KA induced TLE rats. (**A,B**) Rare Nav1.6 is colocalized with the astrocytic marker GFAP in the CA1 and CA3 subregions in the hippocampus in the controls. (**C,D**) Nav1.6 is highly colocalized with the astrocytic marker GFAP in the CA1 and CA3 subregions in the ipsilateral hippocampus at 21 days after-SE in KA induced TLE rats. (**E**,**F**) Note Nav1.6-ir is extensively distributed in the soma and process of GFAP-ir positive cells in the ipsilateral hippocampus at 21 days after-SE in KA induced TLE rats at high magnification. The colocalization ratios of Nav1.6 with GFAP in the hippocampal CA1 and CA3 subregions in KA induced TLE rats and the controls are shown in (**G**,**H**). Data are plotted as mean ± SEM of five animals in each group. SO: stratum oriens; SR: stratum radiatum; SL: stratum lucidum; SP: stratum pyramidale. *P < 0.05, **P < 0.01, significantly different from control animals.

To ascertain the neural cell type in which Nav1.6 is localized, double immunofluorescence staining was employed. Glial fibrillary acid protein (GFAP) is regarded as a prototypical marker for immunohistochemical identification of astrocytes. In the saline-injected hippocampus, GFAP-ir positive astrocytes have small cell bodies with slender processes (Fig. 6A,B). In the ipsilateral hippocampus of rats with SE, severe reactive astrogliosis was observed at 7, 21 and 63 days in the whole injected hippocampus after SE as GFAP-ir positive astrocytes showed enlarged soma size with increased GFAP-ir that are the prominent features of reactive astrocytes (Fig. 6C–F), exhibiting a pronounced astroglial scar at 21 days after SE. Additionally, a number of GFAP-ir positive astrocytes were observed in the contralateral hippocampus of rats with SE, and there were two rats at 21 days after SE showing marked reactive astrogliosis in the contralateral hippocampus. Moreover, the severity of reactive astrogliosis was closely correlated with the severity of SE which is assessed by the number of stage 5 seizures occurring during SE (at 7 days after SE, R^2^ = 0.973, *P* < 0.01; at 21 days after SE, R^2^ = 0.84, *P* < 0.05; at 63 days after SE, R^2^ = 0.933, *P* < 0.01) (Table 1).

Double immunostaining showed that a large amount of Nav1.6-ir was mostly localized in the GFAP-ir positive reactive astrocytes in the ipsilateral hippocampus in post-SE animals, whereas little Nav1.6 is colocalized with GFAP in the control hippocampus (Fig. 6A–D). Nav1.6-ir was not only confined to the hypertrophic soma, but also distributed in the processes of reactive astrocytes (Fig. 6E,F). The co-localization ratios of Nav1.6 with GFAP in the ipsilateral hippocampus in 1 day, 7 days, 21 days and 63 days post-SE animals were significantly higher than those of the contralateral side and the controls, reaching the highest level at 21 days after SE (Fig. 6G,H). Moreover, the number of Nav1.6-ir positive cells was strongly correlated with the severity of reactive astrogliosis (R^2^ = 0.899) (Fig. 5C).

**Figure 7:**
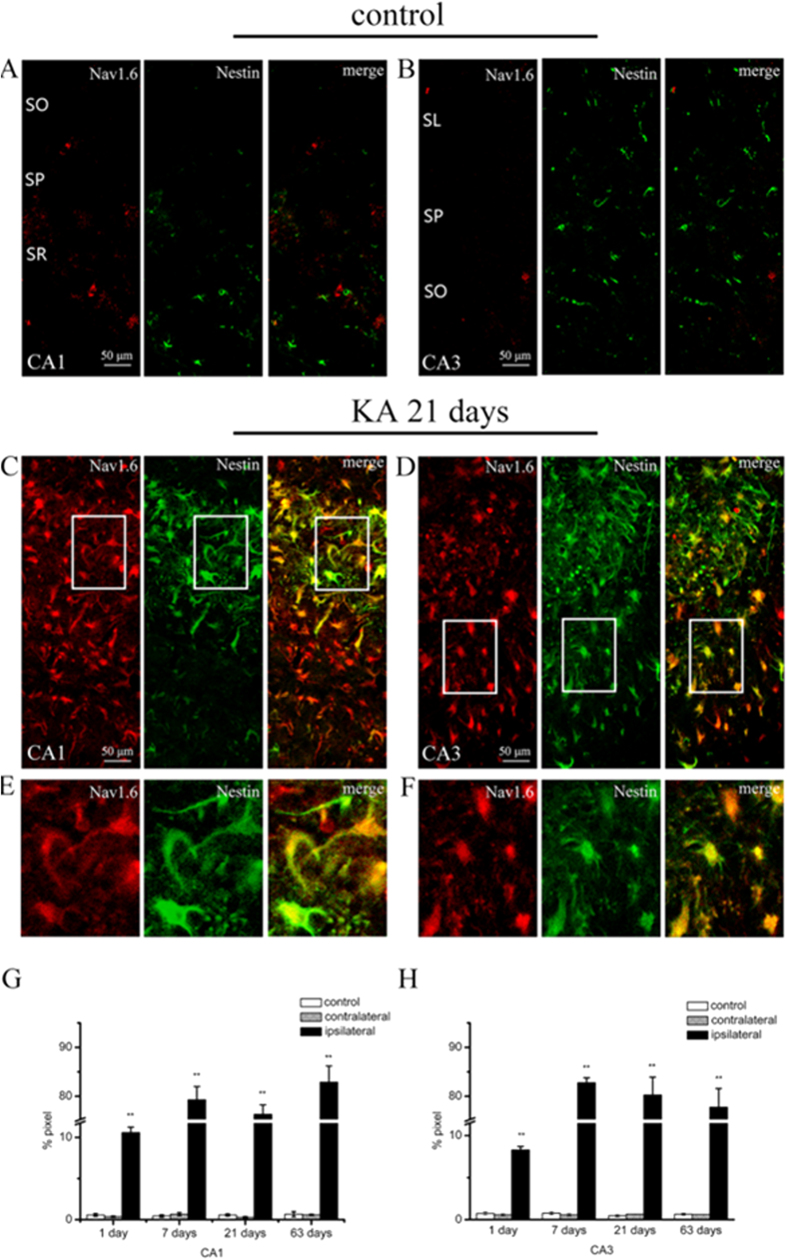
Colocalization of Nav1.6 (red) with nestin (green) in the hippocampus in KA induced TLE rats. (**A,B**) Rare Nav1.6 is colocalized with nestin in the CA1 and CA3 subregions in the hippocampus in the controls. (**C,D**) Nav1.6 is highly colocalized with nestin in the CA1 and CA3 subregions in the ipsilateral hippocampus at 21 days after-SE in KA induced TLE rats. (**E,F**) Note Nav1.6-ir is extensively distributed in the soma and process of nestin-ir positive cells in the ipsilateral hippocampus at 21 days after-SE in KA induced TLE rats at high magnification. The colocalization ratios of Nav1.6 with nestin in the hippocampal CA1 and CA3 subregions in KA induced TLE rats and the controls are shown in **G** and **H**. Data are plotted as mean ± SEM of five animals in each group. SO: stratum oriens; SR: stratum radiatum; SL: stratum lucidum; SP: stratum pyramidale. *P < 0.05, **P < 0.01, significantly different from the control animals.

Nestin, a type VI intermediate filament protein, is found to be expressed in reactive astrocytes in the hippocampal lesions. Similar with the results of the colocalization of Nav1.6 with GFAP, Nav1.6-ir was highly localized in the nestin-ir positive cells in the ipsilateral hippocampus in 1 day, 7 days, 21 days and 63 days post-SE animals, whereas there was little Nav1.6 expressed in the nestin-ir positive cells in the control hippocampus (Fig. 7A–F). The co-localization ratios of Nav1.6 with nestin in the ipsilateral hippocampus were sustainably increased at 1 day, 7 days, 21 days and 63 days after SE compared with the contralateral side and the control hippocampus (Fig. 7G,H).

**Figure 8:**
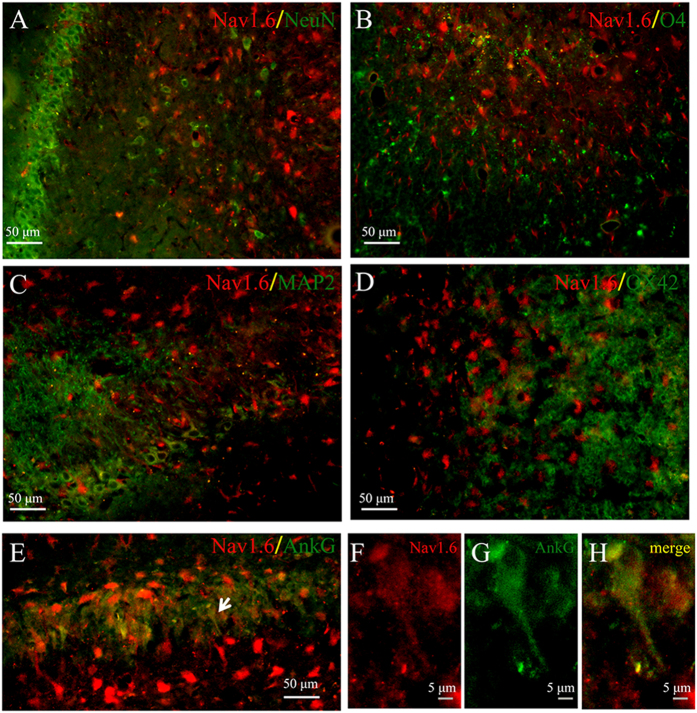
Colocalization of Nav1.6 (red) with NeuN, MAP2, O4, OX-42 or Ankyrin G (green) in the CA3 subarea of the ipsilateral hippocampus at 21 days after-SE in KA induced TLE rats. Nav1.6 is seldom co-expressed with the neuronal marker NeuN (**A**) or MAP2 (**C**), oligodendrocyte marker O4 (**B**), and microglia marker OX42 (**D**). A small amount of Nav1.6 is co-localized with Ankyrin G (**E–H**).

Ankyrin G (AnkG), an integral transmembrane protein of the axolemma, is localized at axon initial segments, and nodes of Ranvier of myelinated axons. We also double-immunolabeled Nav1.6 with AnkG in KA induced post-SE animals. A little Nav1.6-ir was co-localized with AnkG in the ipsilateral hippocampus after SE in KA induced post-SE animals (Fig. 8E–H). Furthermore, other molecular markers of neural cells including NeuN, MAP2, O4, OX42 were used for identification of neuron, microglia, oligodendrocyte. We observed that little Nav1.6-ir was localized in the three types of neural cells in KA induced post-SE animals (Fig. 8A–D).

### Rare expression of Nav1.6 in the PTZ-kindled hippocampus

**Figure 9:**
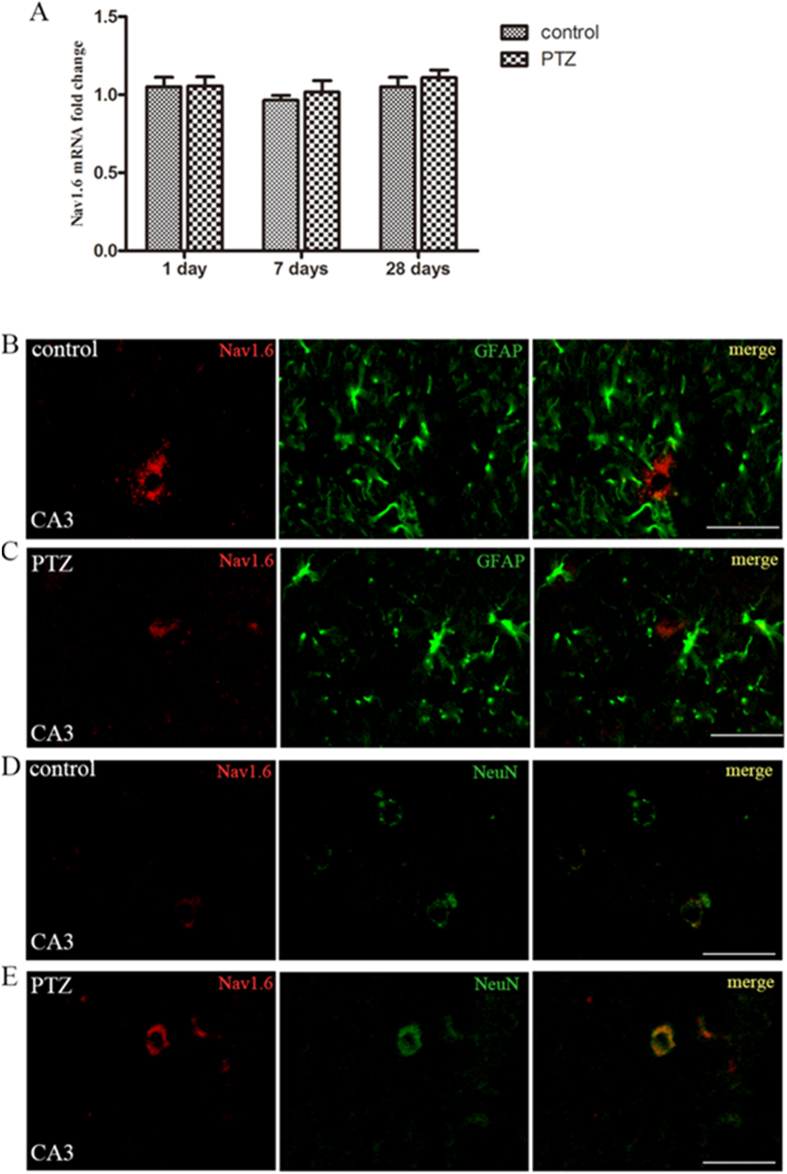
Expression of Nav1.6 in the hippocampus in PTZ-kindled animals. (**A**) Real-time PCR analysis for Nav1.6 expression in the hippocampus in PTZ-kindled animals and the control groups. Each column represents mean ± SEM of five animals in each group. *P < 0.05, **P < 0.01, significantly different from the control animals. (**B–E**) A small amount of Nav1.6-ir is observed in the stratum pyramidale in the hippocampus in PTZ-kindled animals and the controls. Nav1.6-ir (red) is not co-localized with GFAP-ir positive cells (green) both in the controls and PTZ-kindled rats (**B,C**). Nav1.6-ir (red) is located in NeuN-ir positive cells (green) both in the controls and PTZ-kindled rats (**D,E**).

Quantitative real-time PCR showed that the expression of Nav1.6 at mRNA level in hippocampus in fully kindled PTZ was not significant difference from the sham -kindled controls (Fig. 9A). Moreover, immunofluorescence did not show reactive astrogliosis in the hippocampus in kindling animals (Fig. 9B–E). A small amount of Nav1.6-ir was detected in the stratum pyramidale (SP) of the hippocampal CA area both in PTZ-kindled animals and the controls (Fig. 9B–E). Furthermore, there were no significant differences in the expression of Nav1.6 in the hippocampus between PTZ-kindled animals and the controls. NeuN is a widely used marker to label neurons. Interestingly, Nav1.6-ir was co-localized with NeuN rather than GFAP in the hippocampus in the kindling animals (Fig. 9B–E).

## Discussion

In the present study, VGSCs subtype Nav1.6 is highly expressed in the ipsilateral hippocampus of rats experiencing SE induced by KA. The expression of Nav1.6 initiates early after the initial SE, reaches the highest level at the latent stage, and then slightly decreases at the chronic stage in the ipsilateral hippocampus in KA-induced post-SE TLE model. Moreover, the majority of Nav1.6-ir is located in reactive astrocytes rather other in neurons. However, Nav1.6 expression is not changed and selectively distributed in neurons rather than astrocytes in the hippocampus in PTZ-kindled animals.

The process of epileptogenesis is classically considered to occur in three stages: first, the occurrence of an initial injury; second, a ‘latent’ period during which seizures do not occur; and third, chronic epilepsy is established. The latent phase is thought to be a critical period during which changes occur in gene, cellular and circuit-levels, resulting in a transform of normal brain into epileptic brain^6^. Early work has reported the increased expression of Nav1.6 during the latent period in post-SE animal induced by electrical stimulations^12^. In agreement, our present study shows that Nav1.6-ir in the ipsilateral hippocampus in post-SE animals is persistently increased during the latent and chronic phases, and has the highest level at the latent phase. Furthermore, only rats that previously experienced SE in our study exhibit the increase expression of Nav1.6, and this increase is profoundly seizure intensity-related. Moreover, it has been reported that reduced expression of Nav1.6 and inhibition of Nav1.6 activity can suppress the neuronal hyperexcitability^12^,^15^,^24^. Thus, the increased Nav1.6 expression could be an important molecular alteration in the course of epileptogenesis.

Additionally, our results show that the majority of Nav1.6 immunoreactivity is mostly localized in reactive astrocytes rather than neurons in the ipsilateral hippocampus in post-SE animals. Prior investigations have demonstrated that Nav1.6 was mainly expressed in the axon initial segment (AIS) and the dendrites of the hippocampal CA pyramidal cells^18^,^25^. Nonetheless, astrocytes in primary cultures have been shown to express Nav1.6^26^,^27^. A previous study from Qiao *et al*. has showed that Nav1.6 was expressed in astrocytes, but was persistently decreased during epileptogenesis in the hippocampus in post-SE animals induced by systemic KA injection^14^. The disagreement of Nav1.6 expression with Qiao *et al*. may ascribe to the induction of pronounced reactive astrogliosis in the ipsilateral hippocampus in the intrahippocampal KA injection model. Compared with the intrahippocampal administration of KA, the systemically administered KA models have more high morality^23^. To reduce the morality, status epilepticus induced the systemic injection of KA is thereby earlier halted by anticonvulsants, which could reduce the intensity of seizure activity and affect the development of the hippocampal lesions^23^. Notably, the increase of Nav1.6 expression in our experiments is strongly correlated with the intensity of seizures and the severity of reactive astrogliosis in the hippocampus. Reactive astrogliosis is a prominent cellular event in the development of epileptogenesis, during which astrocytes undergoes sequential changes in morphology, molecular composition, and proliferation^28^. It is generally acknowledged that neurons as the basic functional elements generate action potential and express seizure discharges in nervous system, while astrocytes are unable to generate action potential^29^. However, several lines of evidence have been reported that reactive astrocytes from the seizure focus in primary cultures and acute biopsy slices of human patients with temporal lobe epilepsy were recorded large voltage-dependent Na+ currents and can be stimulated to generate action potential-like responses^30^. Additionally, previous patch-clamp studies have demonstrated voltage-dependent sodium currents elicited from astrocytes in primary cultures, which is transient, show rapid activation and inactivation, and can be inhibited with the VGSCs specific toxin tetrodotoxin (TTX)^31^. Thus, the increased expression of Nav1.6 in reactive astrocytes may contribute to hyperexcitability in the epileptic brain. On the other hand, various lines of evidence have described that the release of glutamate regulated by intracellular Ca^2+^ fluctuations may be involved in epileptiform discharges in animal models and human patients with TLE^28^,^32^. The intracellular Ca^2+^ concentrations were largely mediated by the activity of Na^+^/Ca^2+^ exchange which is driven by the intracellular Na^+^ concentrations^26^,^27^,^33^. It is hence supposed that VGSCs subtype Nav1.6 at high expression levels in reactive astrocytes could represent a new mechanism for modulation of glial-neuronal communication by controlling Na^+^ influx to influence the Ca^2+^ signals in astrocytes, mediating the release of glutamate from astrocytes, subsequently contributing to the generation of synchronized epileptiform activity.

However, the dramatic alterations of Nav1.6 expression in reactive astrocytes were not observed in the hippocampus in the PTZ-kindled animals in this study. Despite the expression of Nav1.6 protein and mRNA has been demonstrated to be selectively increased in hippocampal CA3 neurons in electrical kindled-rats^15^, Nav1.6 in our PTZ-kindled animals is expressed at low levels and selectively distributed in the hippocampal neurons. Post-SE TLE models can closely mimic the pathological process of TLE in humans, especially the progression of hippocampal sclerosis characterized by neuron loss and reactive astrogliosis. However, kindled animals are not associated with hippocampal neurodegeneration because kindling usually leads to no or minimal hippocampal sclerosis^27^,^34^. In accordance to the previous studies, we did not find the obvious hippocampal damage characterized by neuron loss and reactive astrogliosis in the PTZ- kindled animals. In addition, we also observed the increased expression of Nav1.6 in reactive astrocytes in the contralateral hippocampus. Meanwhile, our results show that the expression of Nav1.6 was markedly correlated with the severity of reactive astrogliosis. So, the Nav1.6 expression at low levels may be attributed to the lack of the reactive astrogliosis in the hippocampus in PTZ-kindled animals. Thus, these findings further reveal that the altered expression of Nav1.6 may be closely correlated with the process of reactive astrogliosis in the hippocampus during epileptogenesis.

Currently, multiple anti-epileptic drugs (AEDs) generally exert their anti-seizure activities through targeting ion channels as well as excitatory and inhibitory neurotransmission. Most studies focusing on the mechanisms of the action of AEDs on these molecules in neurons often neglect the effects of AEDs on these molecules in astrocytes. Thus, these findings may promote the understanding of the roles of reactive astrocytes in the epileptogenesis and the pharmacoresistance in epilepsy. A new study has shown that reactive astrogliosis can cause the occurrence of spontaneous seizures in the absence of other pathologies and without BBB breach or significant inflammation^35^. Therefore, targeting VGSCs expressed in astrocytes could be a promising therapeutic strategy for epilepsy. Further work will be performed to determine whether altered expression of Nav1.6 in reactive astrocytes can contribute to neuronal hyperexcitability and the development of epilepsy.

## Methods

### Animal preparation

All animal procedures were approved by the Institutional Animal Care and Use Committee (Department of Laboratory Animal Science, Shanghai University) and carried out in full accordance with institutional animal care use protocols of Laboratory Animals of Shanghai University. Male Sprague Dawley rats (Shanghai experimental animal center, Chinese Academy of Sciences) weighing 210–240 g were used for the experiments. Animals were kept in individual cages with water and food available ad libitum. The animal room was maintained at 21–25 °C on a 12 h light/dark cycle.

#### KA induced post status epilepticus model

The KA induced post-SE rats were generated according to the protocol of Stephan Loacker *et al*.^36^. The rats were anesthetized and maintained with a gas mixture of 98.5% air and 1.5% isoflurane, then positioned in a stereotaxic instrument (Stoelting, Wood Dale, IL). After a small craniotomy and opening of the dura, KA (0.5 μg in 2 μl saline, pH 7.4), or saline (2 μl) for control, was injected into the CA3 region of right hippocampus at the following coordinates of Paxinos (2004) altas (4.0–4.2 mm posterior to bregma, 4.1–4.3 mm lateral to midline, and 3.9–4.1 mm below the cortical surface) at a rate of 0.2 μl/min using a microprocessor-controlled syringe pump (Stoelting, Wood Dale, IL). The needle was kept in place for 10 min. Rats were observed and monitored by video/ EEG recording. For EEG recording, electrode was implanted stereotaxically into the right hippocampus at the following coordinates: AP: −5.2 mm, ML: 4.4 mm and DV: 5 mm from bregma and dura surface according to Paxinos (2004). Seizure activities were scored according to Racine’s scale (stage 1–5 seizures). SE was defined by continuous motor seizure activities corresponding to several stage 3 seizures and at least one stage 5 seizure or several stage 4 seizures for at least 2 h. Diazepam (5 mg/kg) was used to increase survival after 4 h of SE. The number of stage 5 seizures occurring over a 5 h period following KA injection was counted to estimate the severity of SE. Spontaneous seizures were continuously monitored for 84 h/week (12 h/day, 7 day/week) by video recording from week 1 to 9 after KA injection. Animals were killed at 1, 7, 21, 63 days after KA injection for quantitative real-time PCR and immunofluorescence analysis, respectively.

#### PTZ induced kindling model

Animals were intraperitoneally injected with 1% pentylenetetrazole (PTZ) (diluted in saline, pH 7.4, 35 mg/kg, Sigma) once daily according to previously published method^37^. The control group received saline injections in an identical manner. Rats were observed and video-tracked for 30 min after injections for the occurrence of behavioral seizures. The severity of seizures was rated according to Racine’s scale (stage 1–5 seizures). Rats exhibiting three subsequent stage 4 seizures were stated fully kindled.

### Extraction of total RNA and quantitative real-time PCR

Rats were anaesthetized using 10% chloral hydrate and killed by cervical displacement. Brains were removed for total RNA extraction. The RNA extraction kit (RNAiso Kit, Takara) was used according to the manufacturer’s protocol for the extraction of total RNA from hippocampus. Genomic DNA were removed by recombinant DNAse I (RNAse-free, Takara) treatment. The concentration and purity of RNA were quantitated by UV absorbance at 260 nm using NanoDrop spectrophotometer (Thermo Fisher Scientific). Complementary DNA (cDNA) were synthesized using a reverse transcription kit as described by the manufacturer. The specific primer sequences for Nav1.6 were forward 5′-AGGATGTTAGCAGCGAATCAGACC-3′ and reverse 5′-GGAGCTGGTATCGTCCAGTTTATC-3′. The primers for reference gene were as follows: (1) beta-actin forward 5′-CGCGAGTACAACCTTCTTGCAG -3′ and reverse 5′-TATCGTCATCCATGGCGAACTGG -3′; (2) GAPDH forward 5′-GCAAGTTCAACGGCACAGTCAAG-3′ and reverse 5′- CGACATACTCAGCACCAGCATCAC-3. Quantitative real-time PCR was performed using Brilliant SYBR Green QPCR Master Mix (TaKaRa, Japan). Amplification and melting curves were recorded using an ABI 7500 system (Bio-Rad, USA). The 2^−ΔΔCt^ method for relative quantification was used to calculate the relative level of Nav1.6 in the different time courses following KA and PTZ injection. All results were analyzed by GraphPad Prism 5.0 software.

### Immunofluorescence

Rats were deeply anesthetized with 10% chloral hydrate and fixed *in situ* by transcardial perfusion with 4% paraformaldehyde (PFA) in 0.1 M PBS (pH = 7.4). Brains were removed and postfixed for 2 h in 4% PFA and then placed in 30% sucrose in 0.1 M PBS until sink down to the bottom of container for cryoprotection. Cryosections of 20 μm for immunohistochemistry were prepared. Sections were firstly incubated in 0.2 mg/ml pepsin (Biotech Well) at 37 °C for 10–15 min according to the procedure developed by Lorincz^25^ and then washed with 0.01 M PBS 3 times for 5 min each. Sections were incubated in 5% normal goat serum (Gibco) containing 0.5% Triton X-100 for 30 min and then incubated in primary antibody in 5% normal goat serum containing 0.5% Triton X-100 overnight at 4 °C. Primary antibodies used included polyclonal rabbit anti-Nav1.6 (1:600, Cat.No: ab65166, Abcam; 1:200, Cat.No: #ASC-009, Alomone), monoclonal mouse anti-GFAP (1:500, Cat.No: #3670, Cell Signaling Technology), monoclonal mouse anti-nestin (1:200, Cat.No: ab6142, Abcam), monoclonal mouse anti-MAP2 (1:500, Cat.No: ab11267, Abcam), monoclonal mouse anti-Ankyrin G (1:500; Cat.No: NB20, Merck), monoclonal mouse anti-OX42 (1:200, Cat.No: sc-53086, Santa Cruz Biotechnology) and monoclonal mouse anti-O4 (1:500, Cat.No: #MAB1326, R&D Systems). Sections were then washed with 0.01 M PBS 3 times for 5 min each and incubated in secondary antibodies including Alexa Fluor^®^ 594-conjugated goat anti-rabbit (1:400, Jackson ImmunoResearch Laboratories) and FITC-conjugated goat anti-mouse (1:200, Jackson ImmunoResearch Laboratories) in darkness. Finally, the sections were coverslipped with 50% glycerin. Images were collected with a Nikon fluorescence microscope. Specificity of the immunoreactions for Nav1.6 was tested as described below. Two antibodies raised against different epitopes were used in the immunofluorescence experiment. The two antibodies recognized and stained an identical pattern of cellular morphology and distribution in the brain sections that is similar with previous reports^18^,^17^. Moreover, the preabsorption test was performed on the brain sections. In brief, the primary antibody Nav1.6 was incubated with a tenfold amount of the synthetic peptide (Acession O88420, Alomone) overnight at 4 °C. The brain sections were incubated with the immunizing peptide absorbed antibody according to the protocol described above. All staining of Nav1.6 was abolished in the brain sections when the the immunizing peptide preabsorbed antibody was used.

### Tissue analysis

Images were captured with a Zeiss LSM 880 confocal microscope operating under identical gain settings with sequential scanning to avoid fluorescence bleed-through, and saved as 16-bit TIFF files. Images from identical regions and layers of hippocampus in control, KA and PTZ rats (4–6 animals for each group) were processed in parallel. Images were integrated and processed to enhance contrast in the figures in Nikon software (NS-Elements BR 4.2.00) with identical settings for the different conditions. For quantification of Nav1.6-immunoreactivity (ir) positive cells and the co-localization ratios between Nav1.6 and cell markers in the hippocampal regions, the coronal sections including the entire hippocampal region assigned to levels from 2.4 to 5.6 mm from bregma according to Paxinos (Paxinos, 2004) per animal were acquired for each group. The number of Nav1.6-ir positive cells was counted in an 800 μm × 300 μm grid laid over the hippocampal subareas of CA1, CA3 and hilus, respectively, in seven sections by using Image-Pro Plus software 6.0. The co-localization ratios between Nav1.6 and other proteins including GFAP, nestin in the hippocampal subareas of CA1 and CA3 in 6 continuous sections near the KA injection site were also analyzed by using Image-Pro Plus software 6.0. The severity of astrogliosis was estimated to score GFAP immunoreactivity, grading into none (−), mild (+), moderate (++) or severe (+++) as previously described^38^.

### Statistical analysis

SPSS 22.0 was used for statistical analyses. Statistical significance was assessed using One-Way ANOVA followed by a post hoc LSD test. Correlations were analyzed using Spearman’s correlation test. All values were expressed as mean ± S.E.M, and a level of 0.05 was considered as a threshold for statistical significance.

## Additional Information

**How to cite this article**: Zhu, H. *et al*. Remarkable alterations of Nav1.6 in reactive astrogliosis during epileptogenesis. *Sci. Rep.* **6**, 38108; doi: 10.1038/srep38108 (2016).

**Publisher's note:** Springer Nature remains neutral with regard to jurisdictional claims in published maps and institutional affiliations.

## Figures and Tables

**Table 1:** The correlative analyses of astrogliosis and the severity of SE.

Case		Number of stage 5 seizures	GFAP+ astroglia	Regression analysis
Controls	R1	0	−	
	R2	0	−	
	R3	0	−	
	R4	0	−	
1 day	R1	1	+	R^2^ = 0.775 *P* = 0.225
	R2	3	+	
	R3	0	+	
	R4	6	++	
7 days	R1	0	+	R^2^ = 0.973 *P* = 0.005
	R2	5	++	
	R3	5	++	
	R4	10	+++	
	R5	13	+++	
21 days	R1	0	++	R^2^ = 0.84 *P* = 0.036
	R2	0	++	
	R3	8	+++	
	R4	7	+++	
	R5	9	+++	
	R6	11	+++	
63 days	R1	0	++	R^2^ = 0.933 *P* = 0.007
	R2	0	++	
	R3	0	++	
	R4	10	+++	
	R5	11	+++	
	R6	13	+++	

The severity of SE was assessed by the number of stage 5 seizures for rats experiencing SE at 7 days, 21 days and 63 days after SE; The severity of astrogliosis was estimated to score GFAP immunoreactivity, grading into none (−), mild (+), moderate (++) or severe (+++).
